# The power to detect artificial selection acting on single loci in recently domesticated species

**DOI:** 10.1186/1756-0500-3-232

**Published:** 2010-08-26

**Authors:** Sten Karlsson, Thomas Moen

**Affiliations:** 1 NOFIMA Marine, Arboretveien 6, N-1432 Ås, Norway; 2 Aqua Gen AS, Postboks 1240, 7462 Trondheim, Norway; 3 CIGENE, Arboretveien 6, N-1432 Ås, Norway

## Abstract

**Background:**

An increasing number of aquaculture species are subjected to artificial selection in systematic breeding programs. Rapid improvements of important commercial traits are reported, but little is known about the effects of the strong directional selection applied, on gene level variation. Large numbers of genetic markers are becoming available, making it feasible to detect and estimate these effects. Here a simulation tool was developed in order to explore the power by which single genetic loci subjected to uni-directional selection in parallel breeding populations may be detected.

**Findings:**

Two simulation models were pursued: 1) screening for loci displaying higher genetic differentiation than expected (high-F_ST _outliers), from neutral evolution between a pool of domesticated populations and a pool of wild populations; 2) screening for loci displaying lower genetic differentiation (low-F_ST _outliers) between domesticated strains than expected from neutral evolution. The premise for both approaches was that the isolated domesticated strains are subjected to the same breeding goals. The power to detect outlier loci was calculated under the following parameter values: number of populations, effective population size per population, number of generations since onset of selection, initial F_ST_, and the selection coefficient acting on the locus. Among the parameters investigated, selection coefficient, the number of generation since onset of selection, and number of populations, had the largest impact on power. The power to detect loci subjected to directional in breeding programmes was high when applying the between farmed and wild population approach, and low for the between farmed populations approach.

**Conclusions:**

A simulation tool was developed for estimating the power to detect artificial selection acting directly on single loci. The simulation tool should be applicable to most species subject to domestication, as long as a reasonable high accuracy in input parameters such as effective population size, number of generations since the initiation of selection, and initial differentiation (F_ST_) can be obtained. Identification of genetic loci under artificial selection would be highly valuable, since such loci could be used to monitor maintenance of genetic variation in the breeding populations and monitoring possible genetic changes in wild populations from genetic interaction between escapees and their wild counterpart.

## Findings

### Context

Massive parallel sequencing/re-sequencing technologies have already provided thousands or even tens of thousands of DNA markers for a number of species, while the genotyping of such numbers of markers is becoming routine due to microarray-based genotyping technologies. The possibilities offered by these developments have already been exploited in order to identify loci under natural selection through genome-wide scans [1]. Some studies have focused on selection due to domestication selection of livestock- (e.g. [2]) and plant species (reviewed in [3]). Only a very limited number of studies have targeted signatures of selection in the context of modern breeding programmes [4]. Such studies could, however, be useful in order to increase our understanding of the locus-level consequences of modern artificial selection. To what extent does, for example, artificial selection lead to significant changes in allele frequency at individual loci, and (implicitly) how likely is it that functional genetic variation may be lost due to artificial selection? The existence of several nearly isolated breeding populations, sharing the same breeding goals, provides opportunities for identifying parallel changes between populations. For aquaculture species only a few generations have passed since selective breeding began, possibly limiting the statistical power to detect selection at single loci. In this study, we wanted to estimate the power to detect selection at single loci as a function of effective population sizes, number of parallel populations, number of generations since onset of selection, selection intensity, and the initial genetic distance between populations.

### Computational methods

Two different methods for detecting selection were considered: 1) detection of loci with lower-than-expected values of F_ST _between selectively bred aquaculture populations (hereafter referred to as farmed populations), and 2) detection of loci with higher-than-expected values of F_ST _between a pool of farmed populations and a pool of wild populations. For both methods, the power to detect selection was estimated by simulating a single, bi-allelic locus both in the absence and presence of selection. The simulation program was written in Python (v2.6), utilising simuPop, a library for general-purpose, individual-based, forward-time population genetics simulation [5]. The code may be found in Additional files: low_fst.txt and high_fst.txt. The parameter values were chosen to be relevant for populations of Atlantic salmon in Norway, the focus of our own research, but should match a wide range of aquaculture species. With some exceptions, the Atlantic salmon breeding programmes share the following features: 1) they have been running for 10 or fewer generations, 2) each breeding programme has four parallel year classes, 3) the populations are more or less isolated with little or no gene flow between year classes, and 4) effective population sizes typically lie in the range of 30-50 ([6], Karlsson *et al*, unpublished data). The breeding programmes were once established from different sets of Norwegian rivers, with some overlap between the different sets [7]. F_ST _values between wild Norwegian populations have been found to lie around 0.05 (allozymes [8,9], microsatellites [10-12]). (These results were backed up by our own data on four wild populations genotyped for 12 microsatellite loci and 13 wild populations genotyped for 4514 SNP loci (unpublished)). On this background, our default simulated data set consisted of 10 closed farm populations (low-F_ST _outlier approach) or 10 closed farm populations and 10 wild populations (high-F_ST _outlier approach), each population having an effective population size of 50. Specifically, we assumed that (directional) selection is only occurring in the breeding programmes and that this selection is leading to convergent evolution among different breeding strains. In an evolutionary context we are thus interested in detecting low-F_ST _outlier loci, that will appear as low-F_ST _outliers when only different breeding strains are being studied, but as high-F_ST _outlier loci when a pool of breeding strains are compared with a pool of wild populations (where no selection is occurring). From now on these different approaches will be referred to as Low- and High-F_ST _outlier approaches, respectively. The base populations of farmed populations were assumed to be drawn from different rivers, so that F_ST _between farmed population at generation 0 (base population) would be similar to F_ST _between wild populations (default = 0.05). Parameter values (Ne, number of populations, and start F_ST_) were altered one at a time in order to assess the impact of the parameter on experimental power.

### Algorithm

Two different approaches for the detection of outlier loci were investigated. The first approach was based on the detection of loci displaying lower-than-expected (under a null hypothesis of no selection) F_ST _values between farmed strains. The second approach was based on the detection of loci displaying higher-than-expected values of F_ST _between a pool of farmed populations and a pool of wild populations. For both approaches, a single bi-allelic locus was simulated with and without selection.

#### Low-F_ST _outlier approach

In each of 1000 iterations, a single overall allele frequency was first drawn randomly from a uniform distribution between 0 and 1. N_pop _populations, each consisting of N_e _animals with a single diploid locus, were then formed. Half of the individuals were designated as males, the other half as females. Genotypes were assigned randomly to individual animals, given the overall allele frequency. Next, random mating was simulated in each population for a number of generations, until the F_ST _value between populations reached the wanted level for initial F_ST _(F_ST(0)_). Following this initial phase, random mating with (alternative hypothesis) or without (null hypothesis) selection was applied for N_gen _generations; selection was applied by defining different fitness values for the different genotypes (assuming no dominance). At the end of each iteration, F_ST _between populations [13] was calculated. This process was iterated 1000 times without selection in order to generate a distribution of F_ST _under the null hypothesis, and 1000 times with selection in order to generate a distribution of F_ST _under the alternative hypothesis. Finally, the power to detect outlier loci was calculated. The power was defined as the fraction of the F_ST _-distribution generated under the alternative hypothesis (i.e. under selection) that was lower than the 5% percentile of the F_ST _-distribution generated under the null hypothesis (i.e. without selection). The Python code can be found in Additional file 1.

#### High-F_ST _outlier approach

In each of 1000 iterations, a single overall allele frequency was first drawn randomly from a uniform distribution between 0 and 1. N_pop _* 2 populations, each consisting of N_e _animals with a single diploid locus, were then formed. Half of the individuals were designated as males, the other half as females. Genotypes were assigned randomly to individual animals, given the overall allele frequency. Random mating was simulated in each population for a number of generations, until the F_ST _value between populations reached the wanted level for initial F_ST _(F_ST(0)_). The populations were then split into two sets of equal size, representing farmed and wild populations. For the farmed populations, random mating with (alternative hypothesis) or without (null hypothesis) selection was simulated for N_gen _generations. For the wild populations, random mating without selection was simulated for N_gen _generations, but the size of each population was first increased to 500 in order to minimise the effect of drift in wild populations. At the end of each iteration, the populations were merged into one farmed and one wild 'metapopulation' and F_ST _between these metapopulations was calculated. This process was iterated 1000 times without selection in order to generate a distribution of F_ST _under the null hypothesis, and 1000 times with selection in order to generate a distribution of F_ST _under the alternative hypothesis. Finally, the power to detect outlier loci was calculated. The power was defined as the fraction of the F_ST_-distribution generated under the alternative hypothesis (i.e. under selection) that was higher than the 95% percentile of the F_ST_-distribution generated under the null hypothesis (i.e. without selection). The Python code can be found in Additional file 2.

### Testing

**Figure 1:**
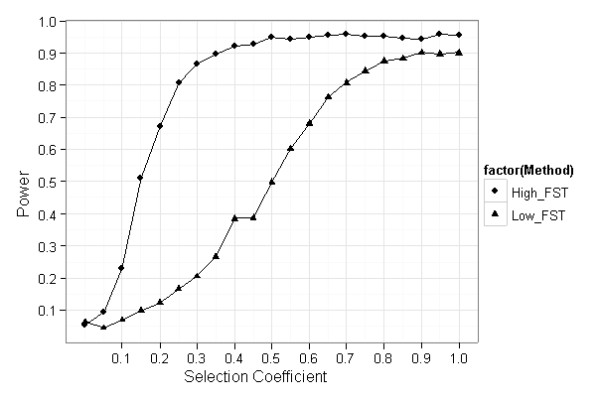
**Comparison between low-F_ST _and high-F_ST _outliers approaches for detecting non-neural loci in domesticated populations**. Power to detect low-F_ST _outliers and high-F_ST _outliers as a function of selection coefficient. Effective population size is 50. Number of populations is 10. Number of generations is 10. Initial F_ST _is 0.05. Number of iterations is 1000.

With default parameter values, the power to detect non-neutral loci among breeding populations (low-F_ST _outliers) was found to be very low, except for extremely large selection coefficients, while relatively small or moderate selection coefficients were found to be sufficient for detecting non-neutral loci, when comparing farmed and wild population (high-F_ST _outliers) (Figure 1).

**Figure 2:**
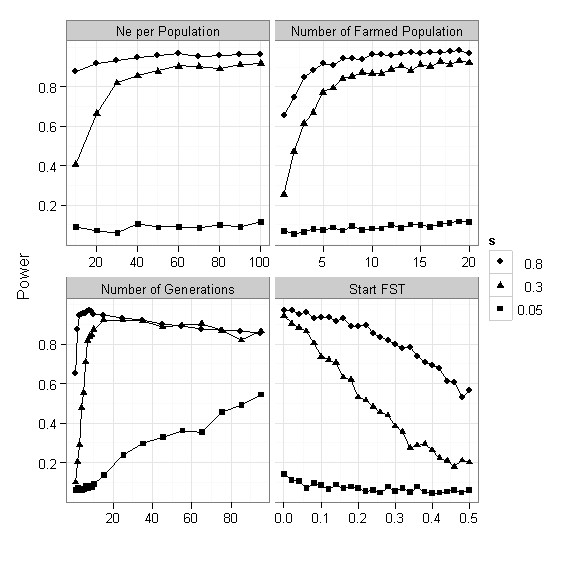
**Power to detect high-F_ST _outliers**. Power to detect high-F_ST _outliers as a function of effective population size (Ne per Population), Number of Farmed populations, Number of Generations, and initial F_ST. _Default parameter values are: Ne = 50, Number of populations is 10, number of generations is 10, and initial F_ST _is 0.05. Number of iterations is 1000.

The power to detect high-F_ST _outliers rapidly increased, and was large for moderate and large selection coefficients, when the effective population size, number of populations and number of generation passed reached 40, 5, and 10, respectively. Power and initial F_ST _was negatively correlated, with a rapid decline in power with an increasing initial F_ST. _The power to detect weak selection (s = 0.05) was close to zero regardless of effective population size, number of populations, and initial F_ST_, but increased with an increasing number of generations since the establishment of the breeding populations (Figure 2).

**Figure 3:**
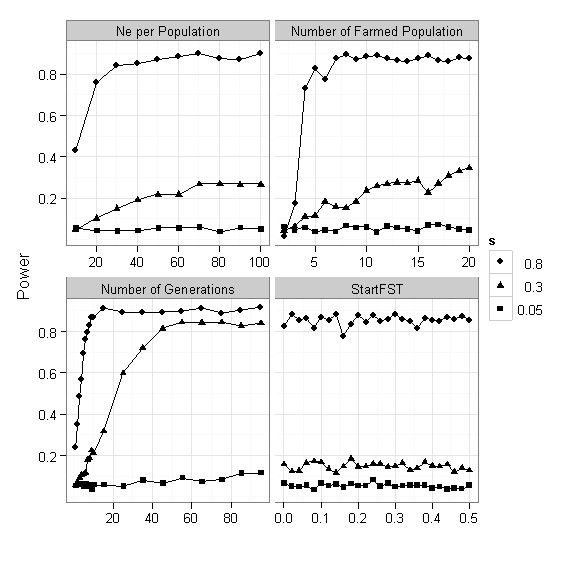
**Power to detect low-F_ST _outliers**. Power to detect low-F_ST _outliers as a function of effective population size (Ne per Population), Number of Farmed populations, Number of Generations, and initial F_ST. _Default parameter values are: Ne = 50, Number of populations is 10, number of generations is 10, and initial F_ST _is 0.05. Number of iterations is 1000.

The power to detect low-F_ST _outliers was not affected by increasing effective population size, or by the initial F_ST_. The largest effect on the power was observed from increasing the number of populations and number of generations (Figure 3).

## Discussion and future development

This study was undertaken as a preparatory step preceding an empirical study seeking to identify (markers for) loci under artificial selection in Norwegian Atlantic salmon breeding programmes. Our motivation for identifying such loci was fourfold: 1) they could potentially serve as universal markers of farmed versus wild Atlantic salmon, 2) they could also be used to elucidate any phenotypic changes occurring in wild salmon as a result of wild-to-farm gene flow, 3) the results could be used to predict to which extent functional loci are likely to be lost due to ongoing artificial selection, and 4) the results could contribute to the identification of loci controlling phenotypic traits in Atlantic salmon. The motivations and the approaches to identify non-neutral loci presented here may also apply to other aquaculture species [14], many of which might escape and interact with their wild counterparts (e.g. common carp [15], tench [16], Atlantic cod [17], Chinook salmon [18], clam [19], Chinese fresh water pearl [20]).

While the infinitesimal model of quantitative genetics assume than complex traits are controlled by many genes with small individual effects, indicating that a study such as this one is futile, experimental results show that some (assumed to be complex) traits are controlled by only a small number of genes. As a notable example, in two independent experiments, both with considerable statistical power, Houston *et al. *[21] and Moen *et al. *[22] identified one and the same QTL controlling the bulk of genetic variation in resistance to the viral disease infectious pancreatic necrosis (IPN) in Atlantic salmon. Many different tests for detecting selection at individual loci have been proposed in the literature (reviewed in [23]). Some of these test for deviation from the neutral expectation of balance between mutation and genetic drift (e.g. Ewens-Watterson homozygosity test [24,25], Tajima's D-test [26], Fu's Fs test [27]), while others test for regions containing long haplotypes of high frequency, indicative of selective sweeps [28,29]. We believe that these two groups of methods to be of little relevance for the current study, since i) the mutation-drift balance is not relevant on the time scale we are addressing (< 10 generations), and since ii) the selection is most likely to have been acting on standing variation, so that long-range extended haplotypes are not likely to be observed ([1] and references therein). While previously developed methods for testing outlier levels of F_ST _between populations or within the same population sampled at different times [30-32] rely solely on observed genetic data for creating an expected F_ST _distribution to which the observed data may be compared, the method presented in this study take advantage of important *prior *knowledge to strengthen the power for detecting outlier loci in a selective breeding context. By utilizing knowledge of known effective population size, number of populations and time since the onset of directional selection, we believe a more accurate (and higher power) expected neutral distribution is obtained. We have therefore taken a more simplistic approach, assuming that selection will be detected through the comparison of differences in F_ST _between populations under (uni-) directional selection or between a selected and an unselected 'metapopulation'. We have further assumed that selection is acting directly upon the locus that is observed, whereas in practice one would more likely be observing a loci closely linked to (and in linkage disequilibrium (LD) with) that locus. As such, we describe best-case scenarios with regard to identifying loci under selection. Power will be lost due to incomplete LD between the observed marker and the locus under selection, but the amount of power lost will vary dependent on species and the marker density used in any given experiment.

The relevance of the power estimates presented here to a real experiment will depend upon how realistic the parameter values are. In addition to the selection coefficient, the number of populations and the number of generations since onset of selection were found to have the largest effect on power. These parameters are usually known for most breeding programs. An accurate estimate of the effective population size may be difficult to obtain unless a full pedigree is available. However, our simulations were robust against varying effective population sizes for Ne larger than 40. The results show that the approaches presented here are not suitable for detecting loci subjected to weak selection, unless many generations (> 75) have passed since the onset of artificial selection. Quantitative Trait Loci (QTL) mapping is used for finding markers linked to commercially important traits; although QTL studies are powerful in linking non-neutral markers to phenotypic trait, the studies are restricted to the study of predefined traits, and are therefore not suitable for screening the whole genome for non-neutral loci influencing any trait under artificial selection. The approaches presented in the present study enable future screening of whole genomes for signatures of artificial selection.

## Competing interests

The authors declare that they have no competing interests.

## Authors' contributions

TM and SK contributed equally in the design of the simulation models and in writing the manuscript. The programming work was done by TM. SK run the simulations and created the figures. Both authors approved of the manuscript.

## Supplementary Material

Additional file 1 **High-Fst**. Python code for simulation of power for high-fst outlier approach Click here for file

Additional file 2 **Low-Fst**. Python code for simulation of power for low-Fst outlier approach Click here for file
